# Constipation

**Published:** 1877-12

**Authors:** 


### *CONSTIPATION.*

Every physician has noticed the air of unconcern with which his patients almost always refer to that condition of the system which is certainly laying the foundation of some of the most painful and fatal of diseases. Constipation becomes alarmingly frequent, as people acquire the means of living luxuriously without severe manual labor. It is still more frequent among persons who become so through ignorance of the penalties that surely follow, sooner or later, a want of attention to the regular demands o nature.

When fecal matter is retained in the rectum more than twenty-four hours it commences its destructive work, notably in two ways. First, it is absorbed into the system, and acts as a blood poison. Second, by its accumulation and impacting in the lower portion of the rectum it interrupts the circulation of the blood in the adjoining parts, and may produce from this cause alone, piles, fistula, inflammation of the bladder, disease of the prostrate gland, or cancer of the rectum. In fact, most of these diseases are developed in this way. To a very great extent we hold our health and happiness, and our lives, in our own hands. If we sacrifice health and life through our ignorance, or indifference, it is only in accordance with the decrees of "eternal justice." From whatever cause, there is, unfortunately, great ignorance in regard to the laws of health among a class of people who are otherwise intelligent, and often educated. How many parents are there who look carefully to the habits of their children, upon which all that will make life desirable for them so much depends? Cathartics and clysters should not be used to relieve constipation. They are certain to aggravate the trouble. Almost any other plan of treatment is preferable to this.

That which has, perhaps, met with most general favor relates to the diet. Certain foods have been regarded as specifics for this complaint, and particularly "Graham bread." This often induces activity of the bowels, but it ruins digestion like other cathartics, in a manner which we have heretofore fully explained in the pages of this JOURNAL. Kneading the bowels has sometimes afforded relief, but it affords no permanent benefit, as a rule. We have, however, found a remedy for constipation and for piles which is always safe, and is attended with no inconvenience or discomfort. We have prescribed it in hundreds of cases, and have never known it to fail in giving relief in a single instance. This remedy is "Nelaton's Suppository." It is prepared and sold by Hall & Ruckel, wholesale druggists, 218 Greenwich street, New York, at 50 cents and $1.00 a box, and is sent free by mail on receipt of price.

### *HEALTH FOOD IN A HOTEL.*

When a strong scientific organization was effected some years ago, to provide purer and better and more wholesome foods than the world had previously enjoyed, we predicted in these pages that the time would come when all hotels patronized by the most intelligent classes would be compelled to supply these better foods to their guests, or lose their business. That prediction is now being fulfilled. The Messrs. Leland, proprietors of one of the best and most popular hotels in New York city, announce "Health Foods from the Health Food Company, 74 Fourth Avenue, New York," on their bills of fare, and inform us that this innovation greatly delights their many guests. They authorize us to say that they will mail copies of said bills of fare to all applicants. Other first-class hotels here and elsewhere, will be compelled to supply these noble foods, and until they do, our friends who live out of the city, and who occasionally visit it, should for their own sakes, patronize the "Sturtevant."

